# Impact of Early (<24 Hours) vs. Delayed (≥24 Hours) Surgery on Hemoglobin Decrease in Patients With Hip Fracture on Direct Oral Anticoagulants: A Multicenter Retrospective Cohort Study

**DOI:** 10.2106/JBJS.OA.25.00334

**Published:** 2026-01-26

**Authors:** T.E.E. Schiepers, H.C. Willems, D.P.J. Smeeing, E. Bosma, D.H.R. Kempen, M.H. Emmelot-Vonk, D. van der Velde, H.J. Schuijt, the OPTIMIZE-DOAC Study Group, J.H. Hegeman

**Affiliations:** 1 Department of Trauma Surgery, St. Antonius Hospital, Utrecht, the Netherlands; 2 Department of Geriatrics, University Medical Center Utrecht, Utrecht University, Utrecht, the Netherlands; 3 Department of Internal Medicine and Geriatrics, Amsterdam UMC Location, University of Amsterdam, Amsterdam, the Netherlands; 4 Department of Trauma Surgery, Rijnstate Hospital, Arnhem, the Netherlands; 5 Department of Trauma Surgery, Martini Hospital, Groningen, the Netherlands; 6 Department of Orthopedic Surgery, OLVG Hospital, Amsterdam, the Netherlands

## Abstract

**Background:**

Surgery in patients with hip fracture on direct oral anticoagulants (DOACs) is frequently delayed because of concerns about bleeding risk. However, evidence supporting such delays remains limited, and institutional practices vary widely. This study aims to determine whether early surgery within 24 hours is associated with a greater perioperative hemoglobin decrease compared with delayed surgery after 24 hours or more in patients with hip fracture on DOACs.

**Methods:**

This multicenter retrospective cohort study included patients with hip fracture aged ≥70 years on DOACs at admission across 5 hospitals from 2018 to 2023. Patients were stratified by time to surgery: <24 hours (early surgery) versus ≥24 hours (delayed surgery). The primary outcome was hemoglobin decrease in mmol/L. Secondary outcomes included a hemoglobin decrease of more than 2 mmol/L, preoperative and postoperative blood transfusion, packed red blood cells administered, postoperative anemia, hospital length of stay, and in-hospital and 30-day mortality. Multiple linear regression and multiple imputation were applied.

**Results:**

Among the 875 patients included, 504 underwent early surgery and 371 underwent delayed surgery. Early surgery was associated with a lower median decrease in hemoglobin levels (0.6 vs. 0.9 mmol/L, p < 0.001); with an adjusted mean difference of -0.25 mmol/L (95% CI, −0.37 to −0.13, p < 0.001). No significant differences were observed in a hemoglobin decrease of more than 2 mmol/L, blood transfusion rates, postoperative anemia, or in-hospital and 30-day mortality. Early surgery was associated with a shorter hospital length of stay median 2 days (95% CI, 2-3; p < 0.001).

**Conclusions:**

Early surgery within 24 hours was associated with a modestly smaller hemoglobin decrease and a shorter hospital length of stay, without an increased blood transfusion rate or mortality rates compared with delayed surgery. These findings suggest that early surgery in patients with hip fracture on DOACs may be safe and potentially beneficial in reducing hospital length of stay.

**Level of Evidence:**

Level III. See Instructions for Authors for a complete description of levels of evidence.

## Introduction

Hip fractures pose a major public health burden in older adults with many having multiple comorbidities, including cardiovascular disease requiring anticoagulation with direct oral anticoagulants (DOACs)^1-5^. DOACs have largely replaced vitamin K antagonists and heparin because of predictable pharmacokinetics and minimal monitoring, offering greater safety and convenience^6-10^. Currently, 12% to 16% of patients with hip fracture use DOACs at admission, and this proportion is rising with the aging population and the growing use of DOACs^11-13^. Although reversal agents are now available, their high cost limits widespread use, and most protocols for hip fracture surgery recommend delaying surgery for 24 to 48 hours after DOAC discontinuation^14,15^. Conversely, delaying surgery to minimize intraoperative bleeding may prolong bleeding from a nonstabilized fracture, potentially offsetting the intended benefit^16-18^. In addition, shorter time to surgery is associated with significantly improved outcomes^19-22^. Consequently, clinical practice varies substantially in the Netherlands: Some hospitals perform surgery within 24 hours from last DOAC intake based on expert consensus, whereas others still require surgical delay. Although retrospective studies suggest early surgery may be safe, a clear consensus has yet to be established, as only 1 single-center study directly compared early and delayed surgery within 24 hours^23-26^. To address this evidence gap, this multicenter retrospective cohort study aims to determine whether early surgery within 24 hours is associated with a greater perioperative hemoglobin (Hb) decrease compared with delayed surgery after 24 hours or more in patients with hip fracture on DOACs.

## Material and Methods

### Study Design

This multicenter retrospective cohort study included 5 level 2 trauma centers in the Santeon network, a consortium aimed at improving care through shared research and best-practice implementation. This study followed the STROBE guidelines^27^.

### Study Population

Patients aged 70 years or older with an isolated Arbeitsgemeinschaft für Osteosynthesefragen (AO) Foundation/Orthopaedic Trauma Association (OTA) 31A or 31B hip fracture undergoing surgery and using DOACs before admission were eligible^28^. Exclusion criteria included pathologic/periprosthetic fractures, interhospital transfers, use of unapproved DOACs by the European Medicines Agency (eg, betrixaban), and vitamin K antagonists. A convenience sample of all eligible patients with complete surgical timing data was used to reflect real-world practice, but no a priori sample size calculation was performed.

### Data Collection

Data from 2018 to 2023 were extracted using the centralized Health Intelligence Platform Santeon (HIPS), enabling uniform automated data collection from electronic health records across all hospitals^29^. Only patients using DOACs at admission were included and data on ineligible patients were not available.

### Definition of Study Groups

Three hospitals adopted protocols permitting hip fracture surgery within 24 hours of last DOAC intake whereas the other 2 retained conservative protocols requiring at least a 24-hour delay. Time to surgery from emergency department (ED) presentation was used as a pragmatic proxy for the DOAC discontinuation interval, because the timing of the last DOAC dose and the reasons for surgical delay were not available retrospectively. This approach was considered justified because surgical timing was primarily determined by institutional protocols. Patients were therefore stratified into 2 cohorts based on their time to surgery from ED presentation: the early surgery cohort (<24 hours) and the delayed surgery cohort (≥24 hours). Perioperative care was comparable across all centers.

### Baseline Characteristics

Baseline variables included age, sex, prior dementia diagnosis (by a geriatrician or general practitioner), body mass index (BMI), estimated glomerular filtration rate (eGFR in mL/min), preoperative Hb (mmol/L), DOAC type (apixaban, edoxaban, rivaroxaban, or dabigatran), American Society of Anesthesiologists (ASA) score, prefracture living situation (home or institutionalized care facility), surgical procedure (hemiarthroplasty, total hip arthroplasty, intramedullary nail, or sliding hip screw), and anesthesia type (general or spinal)^28,30-32^.

### Outcome Measures

The primary outcome was hemoglobin decrease, calculated as the difference between preoperative Hb measured at the ED and the lowest Hb within 24 hours postoperatively. Secondary bleeding-related outcomes included Hb decrease of more than 2 mmol/L, preoperative and postoperative blood transfusion, number of packed red blood cells (PRBC) administered postoperatively (categorized as 0, 1, 2, 3, or 4 or more), and postoperative anemia (defined as Hb < 8.5 mmol/L in male patients or <7.5 mmol/L in female patients within 24 hours postoperatively, in patients without anemia at baseline). Other secondary outcomes were decreased in-hospital and 30-day all-cause mortality, surgery duration (minutes), hospital length of stay (HLOS) (days from ED presentation to discharge or death), and discharge destination (home, rehabilitation center, institutionalized care facility, or hospice). Intraoperative blood loss and postoperative hematoma were excluded because of subjectivity. Wound-related complications (ie, infection or reoperation) were inconsistently captured and could not be analyzed reliably.

### Statistical Analysis

All analyses were performed using RStudio (version 2024.04.1, Posit, PBC)^33^. The level of significance (α) was set at 0.05. Normality of continuous variables was determined using histograms. Normally distributed variables were presented as mean with standard deviation (SD). Nonnormally distributed variables were presented as median with interquartile range (IQR) and analyzed with Mann-Whitney *U* tests. Median differences were estimated using the Hodges-Lehmann estimator. Categorical variables were presented as frequencies and percentages (n, %) and analyzed using the χ^2^ test or Fisher exact test, with effect sizes reported as risk ratios (RR) or odds ratios (OR) with 95% confidence interval (CI). Multiple linear regression was performed on the primary outcome to adjust for potential confounders: age, sex, eGFR, preoperative Hb, DOAC type, ASA score, and surgical procedure. Covariates were selected based on clinical relevance and literature^34-36^. Interaction terms were tested where clinically plausible. Three sensitivity analyses were performed: excluding patients operated on after more than 48 hours after ED presentation and stratifying analyses by surgical procedure and by anesthesia type. No power calculation was performed, and type II error can therefore not be excluded.

### Multiple Imputation

Missing data were handled using multilevel multiple imputation by chained equations (MICE), accounting for hospital-level clustering using the mice package^33^. Predictive mean matching was applied for continuous variables and logistic regression for binary variables (20 data sets, 50 iterations)^37^. Relevant predictors are listed in Appendix List 1. Convergence was verified using trace, density, and strip plots. Key missing variables were ASA score (30%), BMI (22%), and Hb decrease (15%) (Appendix; Supplementary Table 1).

### Ethical Considerations

Ethical approval was granted by the Medical Ethics Committee United (MEC-U; W23.215), which waived informed consent, and local approval was obtained from all participating hospitals. The study complied with the Declaration of Helsinki.

### Level of Evidence

Level 3.

## Results

### Baseline Characteristics

**Fig. 1:**
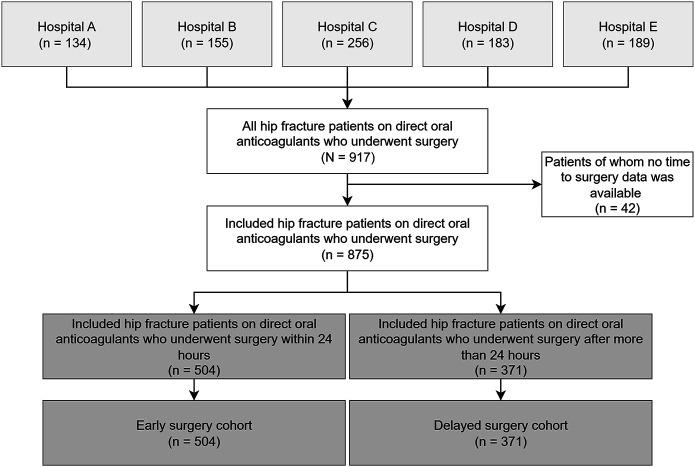
Flow diagram showing patient selection, exclusions, and cohort allocation according to time to surgery (<24 hours versus ≥24 hours).

A total of 917 patients with hip fracture on DOACs were identified; 42 were excluded because of missing data on time to surgery, yielding a study population of 875 patients (504 early surgery cohort, 371 delayed surgery cohort) (Fig. 1). Baseline characteristics were largely comparable between cohorts (Table I). Patients in the early surgery cohort were slightly younger, more often female, had lower ASA scores, and more frequently underwent intramedullary nailing or total hip arthroplasty. Hemiarthroplasty and spinal anesthesia were more common in the delayed surgery cohort. Other variables were generally similar between the 2 cohorts.

**TABLE I:** Baseline Characteristics of Patients With Hip Fracture on DOACs

Baseline Variables	Total N = 875	Early Surgery Cohort n = 504	Delayed Surgery Cohort n = 371
Age (years)	82.3 (5.8)	81.8 (5.8)	83.0 (5.8)
Female sex	568 (65)	335 (66)	233 (63)
Dementia	71 (8)	38 (8)	33 (9)
BMI (kg/m^2^)	25.0 (4.2)	25.0 (4.2)	25.2 (4.2)
eGFR (mL/min)	65.0 (50.0-81.0)	66.0 (49.5-81.0)	65.0 (51.0-81.0)
Preoperative Hb (mmol/L)	7.8 (1.0)	7.7 (1.0)	7.8 (1.1)
DOAC			
Apixaban	304 (35)	163 (32)	141 (38)
Edoxaban	63 (7)	40 (8)	23 (6)
Rivaroxaban	303 (35)	179 (36)	124 (33)
Dabigatran	205 (23)	122 (24)	83 (22)
ASA			
ASA I	3 (0)	2 (0)	1 (0)
ASA II	197 (23)	123 (24)	74 (20)
ASA III	615 (70)	351 (70)	264 (71)
ASA IV	60 (7)	28 (6)	32 (9)
Living situation			
Home	761 (87)	447 (89)	314 (85)
Institutionalized care facility	114 (13)	57 (11)	57 (15)
Surgical procedure			
Hemiarthroplasty	309 (35)	127 (25)	182 (49)
Total hip arthroplasty	100 (11)	86 (17)	14 (4)
Intramedullary nail	386 (44)	241 (48)	145 (39)
Sliding hip screw	80 (9)	50 (10)	30 (8)
Anesthesia type			
General	631 (72)	404 (80)	227 (61)
Spinal	244 (28)	100 (20)	144 (39)
Time to surgery (hours)	21.6 (13.4-37.9)	15.2 (5.6-19.9)	40.4 (30.4-46.8)

ASA = anesthesiologists, BMI = body mass index, and DOAC = direct oral anticoagulant.

### Patient Outcomes

The median Hb decrease was significantly lower in the early surgery cohort (0.6 mmol/L; IQR 0.0-1.4) than in the delayed surgery cohort (0.9 mmol/L; IQR 0.4-1.5), with a median difference of 0.3 mmol/L (95% CI, 0.2-0.4; p < 0.001) (Table II). No significant differences were observed in Hb decrease of more than 2 mmol/L (RR 0.87; 95% CI, 0.70-1.08), preoperative blood transfusion (RR 0.72; 95% CI, 0.50-1.05), postoperative blood transfusion (RR 1.11; 95% CI, 0.97-1.26), PRBC units (OR 1.27; 95% CI, 0.91-1.77), or postoperative anemia (RR 1.02; 95% CI, 0.89-1.15). Similarly, no significant differences were found in in-hospital mortality (RR 0.81; 95% CI, 0.47-1.39; p = 0.548) or 30-day mortality (RR 0.78; 95% CI, 0.54-1.14; p = 0.208). However, early surgery was associated with a significantly shorter HLOS (median 2 days; 95% CI, 2-3; p < 0.001), and a lower likelihood of discharge to a higher level of care (OR 0.64; 95% CI, 0.49-0.83; p = 0.016).

**TABLE II:** Outcomes of Patients With Hip Fracture on DOACs who Underwent Early or Delayed Surgery

Outcome Variable	Early Surgery Cohort n = 504	Delayed Surgery Cohort n = 371	Effect Estimate (95% CI)	p
Hb decrease (mmol/L)	0.6 (0.0-1.4)	0.9 (0.4-1.5)	**0.3 (0.2 to 0.4)**	**<0.001**
Hb decrease of more than 2 mmol/L	44 (9)	43 (12)	RR 0.87 (0.70 to 1.08)	0.200
Preoperative blood transfusion	16 (3)	22 (6)	RR 0.72 (0.50 to 1.05)	0.071
Postoperative blood transfusion	111 (22)	67 (18)	RR 1.11 (0.9 to 1.26)	0.176
Postoperative PRBC administered			OR 1.27 (0.91 to 1.77)	0.581
0 PRBC	393 (78)	304 (82)		
1 PRBC	25 (5)	15 (4)		
2 PRBC	63 (13)	35 (9)		
3 PRBC	9 (2)	8 (2)		
4 or more PRBC	14 (3)	9 (2)		
Postoperative anemia	134 (27)	96 (26)	RR 1.02 (0.89 to 1.15)	0.874
Mortality				
In-hospital mortality	7 (1)	8 (2)	RR 0.81 (0.47 to 1.39)	0.548
30-d mortality	15 (3)	18 (5)	RR 0.78 (0.54 to 1.14)	0.208
Surgery duration (minutes)	96 (77-119)	96 (81-117)	0 (−4 to 4)	0.975
Hospital length of stay (days)	6 (4-9)	8.0 (6-12)	**2 (2 to 3)**	**<0.001**
Discharge destination			**OR 0.64 (0.49 to 0.83)**	**0.016**
Home	130 (26)	66 (18)		
Rehabilitation center	292 (58)	219 (59)		
Institutionalized care facility	73 (14)	74 (20)		
Hospice	2 (0)	4 (1)		

CI = confidence interval, DOAC = direct oral anticoagulant, Hb = hemoglobin, OR = odds ratio, PRBC = packed red blood cells, and RR = risk ratio.

All variables are presented in total amount (percentage rounded to the nearest whole number) or as median values (interquartile range). Percentages for discharge destination do not sum to 100% because in-hospital mortality was not considered a discharge destination. For ordinal outcomes (eg, discharge destination and number of packed red blood cells administered), the odds ratio reflects the odds of being in a higher category in the early surgery cohort. An odds ratio <1 indicates a lower likelihood of a higher category in the early surgery cohort. Bold indicates statistical significance (p < 0.05).

### Multiple Linear Regression

Early surgery was independently associated with a lower Hb decrease (β = –0.25; 95% CI, –0.37 to –0.13; p < 0.001) (Table III). Higher preoperative Hb levels were associated with a greater decrease (β = 0.23; 95% CI, 0.14-0.31; p < 0.001), with a significant interaction indicating this effect was stronger in female patients (interaction term: β = 0.11; 95% CI, 0.00-0.22; p = 0.046). Intramedullary nailing was also associated with a larger Hb decrease compared with hemiarthroplasty (β = 0.46; 95% CI, 0.33-0.59; p < 0.001).

**TABLE III:** Multiple Linear Regression Analysis of Hb Decrease in Patients With Hip Fracture on DOACs

Variable	Reference	β-Coefficient	CI (95%)	p
Constant		−0.75	−1.97 to 0.47	0.230
Early surgery	Delayed surgery	−**0.25**	−**0.37 to -0.13**	**<0.001**
Age (per year)		0.00	−0.01 to 0.01	0.815
Female sex	Male sex	−0.71	−1.57 to 0.16	0.110
eGFR (per mL/min)		0.00	−0.01 to 0.01	0.082
Preoperative Hb (per mmol/L)		**0.23**	**0.14 to 0.31**	**<0.001**
DOAC	Apixaban			
Edoxaban		−0.06	−0.29 to 0.17	0.610
Rivaroxaban		−0.05	−0.19 to 0.08	0.446
Dabigatran		−0.14	−0.30 to 0.01	0.070
ASA score (per unit)		−0.07	−0.18 to −0.04	0.187
Surgical procedure	Hemiarthroplasty			
Total hip arthroplasty		0.05	−0.16 to 0.26	0.672
Intramedullary nail		**0.46**	**0.33 to 0.59**	**<0.001**
Sliding hip screw		0.07	−0.15 to 0.28	0.543
Interaction: Female sex*preoperative Hb		**0.11**	**0.00 to 0.22**	**0.046**

ASA = anesthesiologists, CI = confidence interval, and DOAC = direct oral anticoagulant.

Regression coefficients (β), 95% confidence intervals, and p-values from a multiple linear regression model with Hb decrease as the dependent variable. A negative coefficient indicates lower hemoglobin decrease. Variables were selected based on clinical relevance. Reference categories are indicated. Model fit: R^2^ = 0.173, adjusted R^2^ = 0.161, F = 13.9, and p < 0.001. An interaction term (*) between sex and preoperative Hb was included. The effect of preoperative Hb on Hb decrease is thus sex-specific: for male patients, the coefficient is 0.23, whereas for female patients it is 0.23 + 0.11 = 0.34, indicating a larger apparent decrease in Hb per mmol/L increase in preoperative Hb among women. Bold indicates statistical significance (p < 0.05).

### Sensitivity Analyses

Sensitivity analyses excluding patients who received surgery after more than 48 hours and stratifying by surgical procedure or anesthesia type showed results consistent with the main analyses (Appendix; Supplementary Tables 2–8).

## Discussion

This retrospective cohort study demonstrated that early surgery in patients with hip fracture on DOACs was not associated with a greater Hb decrease than delayed surgery. Interestingly, adjusted analyses showed a modestly lower Hb decrease (−0.25 mmol/L), a difference unlikely to be clinically meaningful^38-40^. No significant differences were observed in secondary bleeding-related outcomes. Early surgery was also associated with a shorter median HLOS of 2 days. These results suggest that surgery within 24 hours in patients with hip fracture on DOACs does not increase perioperative blood loss and may reduce HLOS.

### Comparison with Previous Literature

The modest difference in Hb decrease aligns with previous studies reporting no significant differences between early and delayed surgery^26,38-42^. Reported Hb decreases ranged from 1.2 to 1.8 mmol/L in early surgery and 1.2 to 1.9 mmol/L in delayed surgery^26,41,42^. This may reflect greater preoperative blood loss in delayed surgery patients because of ongoing bleeding from nonstabilized fractures^26^. Unfortunately, no literature reporting regression analyses on Hb decrease were identified. This study also found that higher preoperative Hb was associated with a greater Hb decrease, particularly in females, consistent with hemodilution effects described in literature^40,43^. Secondary bleeding-related outcomes align with studies reporting similar transfusion rates (15%-51%), but a higher postoperative anemia rate of 41.5% than this study (18%-22%)^14,44^. Early surgery was not associated with higher in-hospital (1%) or 30-day (3%) mortality in this study, consistent with Chen et al. who reported no increase in 30-day mortality with 1 to 3 days of DOAC discontinuation.^45^

Early surgery was further associated with shorter HLOS, consistent with recent studies showing 5.9 to 6.0 days for early surgery and 7.1 to 7.6 days for delayed surgery^14,26^. This probably reflects the absence of protocol-driven delays, enabling faster discharge and earlier rehabilitation, which in turn may improve functional recovery^21,22,46^.

Interestingly, intramedullary nailing was independently associated with greater Hb decrease than hemiarthroplasty, likely because of trochanteric fractures bleeding more than femoral neck fractures, where the capsule provides tamponade^18,47^. Unfortunately, fracture type was not recorded by HIPS, thus this hypothesis could not be tested^29^. Finally, although almost all studies used a 48-hour cutoff, this study, like Wang et al., applied a 24-hour threshold, thereby adding evidence supporting the safety of surgery within 24 hours regarding bleeding risk^26^.

### Strengths and Limitations

This study has several notable strengths. It addresses a pertinent clinical dilemma whether surgery within 24 hours in patients with hip fracture on DOACs is safe, applying a stricter threshold than the commonly investigated 48-hour window. It provides new evidence to guide decisions in an area lacking clear guidelines^48^. The use of clearly defined cohorts, robust statistical methods (including multilevel MICE, multiple linear regression, and both unadjusted and adjusted analyses), and consistency across sensitivity analyses enhance the reliability and clinical relevance of the findings.

However, several limitations must be acknowledged. The retrospective design limited access to key clinical variables, notably the timing of last DOAC dose and reasons for delay, which could have underestimated bleeding risk. This study relied on institutional protocols and assumed patient adherence to prescribed regimens, although actual adherence may have varied. Another limitation was the initial 15% missing data in the primary outcome. In addition, the HIPS database did not capture detailed information on comorbidities, fracture type, or functional status, which may have influenced surgical timing, or on wound-related complications or reoperations, which are important clinical outcomes. Data on excluded patients were also unavailable, limiting insight into potential selection bias. Finally, DOAC serum levels at admission were not recorded, although variability in these levels could affect bleeding risk, as acutely ill patients often present with subtherapeutic or supratherapeutic concentrations^49,50^. Future prospective studies are warranted to address these limitations.

### Interpretation, Clinical Relevance, and Future Research

No clinically significant differences in Hb decrease and other bleeding-related outcomes may reflect the rapid offset of DOACs, which could sufficiently diminish anticoagulant effect within 24 hours^51^. However, this interpretation remains limited without data on past DOAC intake, adherence, delay reasons, and DOAC serum levels. Nonetheless, these findings are highly relevant to hip fracture care. Early surgery may improve patient flow, reduce hospital costs, and facilitate earlier mobilization and rehabilitation, key factors for maintaining independence in older adults^52,53^. Although national guidelines for surgical timing in DOAC patients are lacking, this study supports consideration of early surgery protocols in this population. However, prospective studies with more detailed clinical and pharmacologic data are needed before making definitive recommendations. In conclusion, surgery within 24 hours in patients with hip fracture on DOACs was not associated with a greater Hb decrease, indicating the safety of early surgery.

## Appendix

Supporting material provided by the authors is posted with the online version of this article as a data supplement at jbjs.org (http://links.lww.com/JBJSOA/B91). This content was not copyedited or verified by JBJS.

## APPENDIX. The OPTIMIZE-DOAC Study Group

The authors acknowledge the members of the OPTIMIZE-DOAC Study Group: J.H. Hegeman, MD, PhD; H.H. Wijnen, MD; E.R. Flikweert, MD, PhD; F.J.G. Wijdicks, MD, PhD; W.J.M. van der Stappen, MD, PhD; A.H. van der Veen, MD, PhD.
